# Diagnosis of Temporomandibular Disorders Using Thermovision Imaging

**DOI:** 10.1155/2020/5481365

**Published:** 2020-11-17

**Authors:** Monika Machoy, Liliana Szyszka-Sommerfeld, Mansur Rahnama, Robert Koprowski, Sławomir Wilczyński, Krzysztof Woźniak

**Affiliations:** ^1^Division of Orthodontics, Pomeranian Medical University in Szczecin, Powstańców Wielkopolskich Street 72, Szczecin 70-111, Poland; ^2^Department of Oral Surgery, Medical University of Lublin, Karmelicka Street 7, Lublin 20-081, Poland; ^3^Institute of Biomedical Engineering, Faculty of Science and Technology, University of Silesia in Katowice, Bedzińska 39, 41-200 Sosnowiec, Poland; ^4^Department of Basic Biomedical Science, Faculty of Pharmaceutical Sciences in Sosnowiec, Medical University of Silesia, Będzińska Street 39, Sosnowiec 41-200, Poland

## Abstract

Temporomandibular joint dysfunction (TMD) is a chronic disease of various etiologies. Correct TMD diagnosis enables to apply effective treatment and significantly improves the quality of patients' lives. One of the diagnostic methods subjected to evaluation in recent years is thermography, which enables safe, noninvasive, and quick imaging of the temperature distribution of temporomandibular joint-associated tissues. This paper, based on Medline, Dentistry & Oral Sciences Source, Academic Search Ultimate, Medline Complete databases, presents basic information related to thermovision imaging and outlines the direction of research conducted in recent years which fight with difficulties in the interpretation of thermograms that require specialized, dedicated analysis and processing of the obtained images. The problem concerns also no standardized protocol for measuring masticatory muscle temperature.

## 1. Introduction

Temperature is one of the basic state parameters, determining in thermodynamics the mean kinetic energy of the molecules making up a given system. Temperature can be strictly defined only for thermodynamic equilibrium distates, i.e., stable body temperature or its equalization between two bodies. In medicine, a temperature increase (*calor*) is one of the basic, in addition to redness (*rubor*), swelling (*tumor*), and pain (*dolor*), signs of tissue inflammation, defined by Aulus Cornelius Celsus before 50 AD. Since the beginning of the development of medical diagnostics, *calor*, as the most basic and noticeable feature of an ongoing disease process, has been subjected to analysis and the results of interpretation have been objectified according to the possibilities of contemporary science. The first thermometers created around 200 BC in the ancient cradles of culture and science, namely, Byzantium and Alexandria used the phenomenon of thermal expansion of gases to measure temperature. Galileo also used this phenomenon to create his thermoscope around 1600 [1].

During the centuries that followed, there arose new concepts for thermometers and their construction as well as measurement methods, differing in terms of the type of physical phenomena and sensors used. The measurement of the electrical voltage at the contact of two metals by assessing the changing thermoelement resistance was used (thermocouple method). Moreover, diode, liquid, magnetic, resistance, pyrometric, and other thermometers were created [2]. Electronic thermometers, including those based on the detection of invisible energy of the electromagnetic waves of the wavelength ranging from 7 to 14 *µ*m, were developed after mechanical thermometers. The human eye receives only a small part of the electromagnetic spectrum [3]. In addition to visible rays, the spectrum of electromagnetic radiation includes gamma rays, X-rays, ultraviolet rays, infrared rays, microwaves, and radio waves. Infrared radiation (IR) is produced by all objects with temperatures above absolute zero, including warm-blooded living organisms. The rapid development of technology in the field of infrared radiation measurement and its conversion into a visible image led to the emergence of a new technique called thermography [4]. IR emitted or reflected from warm objects is registered by a detector (thermal or photonic). The thermal imaging camera lens focuses infrared radiation on the surface of a matrix consisting of infrared sensors. The matrix sensors react to the absorption of IR radiation by changing one of the system parameters, e.g., pressure, polarization, resistance, and temperature, and then they are transformed into an image. As a result, thermovision provides images reflecting the physiological processes of living organisms by observing the temperature distribution on the external surface of the examined system without the need for any contact [4, 5]. The use of the term “observation of the temperature distribution on the external surface” not only narrows the area of research to the properties of that surface but also has deeper implications, especially if the observed system is a living organism [6]. Thermal heterogeneity, e.g., on the surface of the skin of the face, largely depends on the blood flow and the type of tissue directly underneath it. Thus, the skin surface above the muscle tissue, which is characterized by high metabolic activity, emits more heat radiation than the skin covering the bone or connective tissue. Therefore, thermography visualizes the thermal properties of tissues in a similar way as radiology illustrates their anatomy [4–7].

The advantages of thermography are noninvasiveness, asepsis, which is extremely important in medicine, the lack of ionizing radiation, and the relatively low cost of testing [8], and many specialties were used in medical diagnostics, mainly dermatology—through the possible analysis of changes in skin temperature [9], obstetrics and gynecology [10], neurology [11], oncology [12, 13], pediatrics [14], ophthalmology [15], orthopedics [16], forensic medicine [17], acupuncture medicine [18], cardiology [19], transplantology [20], and dentistry [21]. The advantages of the thermovision have also contributed to the introduction of this method into the diagnosis of temporomandibular dysfunction [4, 22–33].

Temporomandibular disorders (TMDs) are a collective term covering a number of clinical issues affecting the masticatory structures (muscles), the temporomandibular joint (TMJ), and associated tissues. Different types of TMD can be distinguished. Pain-related temporomandibular disorders (TMD-P) are the most prevalent conditions among TMD. The primary manifestations of TMD-P are pain of a chronic nature in the masticatory muscles and temporomandibular joint and pain projection in adjacent structures such as skin and fascia. The other characteristic symptoms include limitations in the range of mandibular motion and crackling joint noises [34, 35]. The pain frequently radiates to the dental arches, molar teeth, ears, temples, forehead, occiput, cervical region of the spine, or shoulder girdle. TMD causes a reduction in mouth opening as well as discomfort and pain during chewing. Among the chronic diseases that cause facial pain, this dysfunction occurs in different decades of life, but mainly in adulthood [36, 37]. The etiology of TMD is multifactorial—occlusal, anatomical, emotional, and behavioral causes are distinguished [38–45]. One of the most frequent symptoms in multifactorial TMD is orofacial pain. Orofacial pain is defined as a pain manifested in the face or oral cavity, including such disorders as TMD, which are a major cause of nonodontogenic orofacial pain [37]. Such pain can affect ears, eyes, and/or throat, producing neck pain, facial pain, and headaches [46]. Pain is an exclusive, complex experience for each person. The nociception depends on factors such as cultural differences, previous pain experience, knowledge, learned behavior, and expectations that may contribute to the individual response to pain [47]. The International Association for the Study of Pain provides the following definition of pain: “an unpleasant sensory and emotional experience associated with actual or potential tissue damage, or described in terms of such damage” [48].

The first attempts of imaging with the TMD thermal imaging camera were made in the mid-90s of the last century. Attempts have been made to analyse and compare the temperature of the temporomandibular joint area with the clinical symptoms of patients [27], thermographic characterization of internal derangement of the temporomandibular joint [49], diagnostic tool for arthralgia of TMJ [50], asymptomatic TMJ [32, 33], and degenerative joint disease [51]. Due to hardware and procedural limitations, these tests can be described as recognizing the topic and possible applications. With the current technological progress and the development of biomedical imaging, it was justified to analyse the latest research evaluating the possibilities of using the latest thermal imaging cameras in the routine diagnosis of a temporomandibular joint.

## 2. Materials and Methods

The article analyses English-language research from the PubMed/Medline, Dentistry & Oral Sciences Source, Academic Search Ultimate, Medline Complete databases. Since the authors wanted to present the latest achievements and the possibility of using thermography in the diagnosis of the temporomandibular joint, articles were selected that were published within no earlier than 5 years. Only the PubMed database gave satisfactory searching results. The inclusion criteria were research and review articles not older than 5 years, focusing on temporomandibular disorders diagnosed with help of thermovision. Exclusion criteria were articles out of date. There are few articles concerning the topic, so the authors could not use strict exclusion criteria. The search phrases were “TMD and thermovision,” “temporomandibular disorders and thermovision,” “TMD symptoms and thermovision,” “TMD and thermal diagnosis,” “temporomandibular disorders and thermal diagnosis,” and “TMD and thermo”.

The end date of the search was June 2020. 18 articles meeting the above criteria were found; they were research articles, carried out on adult patients of both sexes.

### 2.1. Thermovision as a Diagnostic Tool in TMD

**Figure 1:**
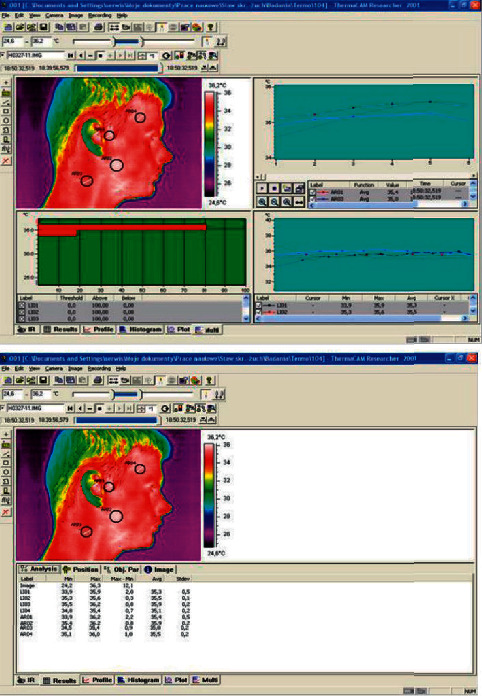
Location of the analysed areas of thermograms in the ThermaCAM Researcher program with measuring window and measured temperature of the exact points.

**Figure 2:**
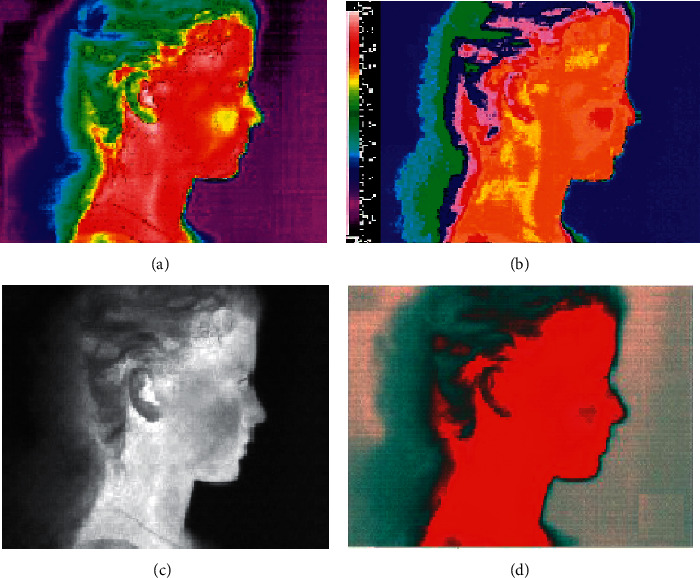
The use of different color scales in the evaluation of thermograms. (a) The “rain” scale enables an accurate analysis of the temperature distribution due to the large range of colors. (b) “Iron” scale enables the blurring of isotherms due to a smaller color range. (c) “Medical” scale enables contrasting color separation in qualitative analysis of thermograms. (d) “Gray” scale enables accurate analysis of temperature distribution based on intensity luminance.

**Figure 3:**
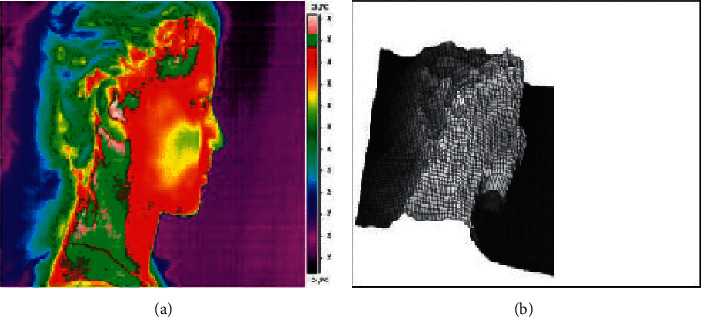
The use of tools: (a) isotherms enabling the determination of areas with the same temperature and (b) 3D visualization algorithm for three-dimensional analysis of the presentation of thermographic results.

In the years 2014–2019, only a few papers were published examining the possibilities of using a thermal imaging camera in the effective diagnosis of temporomandibular joint dysfunction. In the paper by Woźniak et al. [35], sensitivity, specificity, and accuracy of thermography in identifying the degree of dysfunction in patients were assessed. Both facial and neck thermograms were taken using the right and left side projections under constant test conditions. Automatic calibration tools were used during the tests, which enabled to optimize both the level and range of displayed temperatures, and the color palette and the highest contrast in all image areas. Quantitative analysis of thermograms was carried out in selected areas of the face and neck, which were marked with tools in a 1 cm diameter circular area. Due to the possibility of displacement of individual thermograms of the examined subjects, each image was analysed individually and corrections were made if necessary. Despite such a thoroughly conducted methodology and a large study group, based on imaging, it was possible to identify patients only without joint dysfunction in 95.5% of cases. The rest of the results did not allow for precise diagnosis. Thermographic scans from the carried out tests are provided in Figures 1–3 with the consent of the authors.

However, it should be noted that skin surface temperature changes are not a TMD-specific symptom. Also, other diseases including skin diseases can cause a local temperature increase. In the present case, a significant element indicating TMD was the symmetrical occurrence of changes on both sides of the face as well as the specific location of local temperature changes. Nevertheless, thermography alone cannot be treated in this case as a diagnostic tool with high sensitivity and specificity, and thermographic examination should be supplemented with subjective and physical examinations. An additional prognostic factor which was not included in the publication of Woźniak et al. [35] could be the use of dynamic thermovision analysis. In this case, a factor provoking inflammation and/or pain in the temporomandibular joint should be used, and then, the temperature rise should be recorded. Such a factor could be, for example, chewing food.

Also, the research by Barbosa et al. [52] defined the infrared thermography as a rather difficult tool to differentiate TMD because of no significant association between the presence of temperature and pain asymmetry. The main conclusion of the research was that the use of infrared thermography in a day-by-day clinical environment may not be as easy as it seems. The standardization of all protocols needs to ensure that all possible thermal changes related to the image acquisition room and patient's habits do not interfere with the image data acquisition. It is very hard to replicate it in a dental clinic. The image acquisition room must have a perfectly controlled room temperature, and a limited number of staff members should be allowed in the room. The surface temperature of the skin can also be affected by, among others, the day of the menstrual cycle for women, taking hot or spicy dishes, and emotional state. The patient must follow meticulously the professional's instructions for image acquisition, avoiding hot beverages, hot baths, exercises, and other activities or substances that can affect their microcirculation before infrared thermography image acquisition [52].

The thermal imaging camera was also used to visualize TMD in two professional musicians—a violinist and a clarinettist—in whom it was possible to assess temperature changes in various parts of temporomandibular joint-associated muscles, under the influence of occlusal splint therapy [53, 54]. A clinical trial showed internal disorders of both joints, osteoarthritis with prior displacement of the articular disc. The analysed thermograms confirmed the existence of a temperature difference at the level of the front part of the temporal muscle, the joint itself, and the masseter muscle. Thermography also confirmed the effectiveness of occlusive splint therapy by visualizing the temperature drop of given anatomical sites, which indicates a reduction in inflammation that was reported by patients as reduced discomfort. One of the conclusions of the publication was the need to use thermovision techniques in dentistry, which would enable to prevent overloading of certain anatomical structures owing to the early diagnosis of degeneration and its proper monitoring. Therefore, thermographic imaging may not only be a relatively effective diagnostic element, but nevertheless require supplementation with subjective and physical examinations, but may also be used to assess the effectiveness of treatment. Temporomandibular dysfunction therapy should lead to a reduction of inflammation which will result in a change in temperature on the skin surface. However, it should be taken into account that also other drugs not used in TMD may affect the result of such examination. These are, e.g., nonsteroidal anti-inflammatory drugs and glucocorticosteroids. These drugs, used by the patient in other indications, can disturb the temporomandibular joint disorder thermovision image by temporarily reducing inflammation and thus affecting local tissue temperature increase.

In the study by de Melo et al. [55], the possibility of TMD therapy using occlusal splints or low-frequency lasers was compared and evaluated. The clinical results of the treatment were analysed using thermography. Based on them, it was found that both methods were effective in reducing myofascial pain syndrome. Taking into consideration the thermographic data, it was found that the use of lasers can provide more favourable results because lower temperatures within the masseter muscles were visualized in the described study group. The result of the experiment shows the possibility of using a thermal imaging camera as a tool to refine the results of treatment effects already observed clinically. In addition, in the case of nonpharmacological therapies that use physical methods such as laser therapy, thermovision can be useful not only to assess the effectiveness of treatment but also to assess the extent of inflammatory changes. Therefore, it is possible to thermographically precisely mark the inflamed place and to irradiate the laser with that exact place.

The topic of treatment of temporomandibular joint diseases with the help of occlusal splints and evaluation of their effectiveness using thermographic techniques was also discussed in an older, but worth mentioning article by Valentim Adelino Ricardo Bara et˜ al. published in 2010. It was aimed to evaluate the effect of occlusal splint treatment on the temperature of the whole group of muscles that are part of the masticatory system such as masseter (inferior, intermediate, and superior), anterior temporal, digastric, and trapezius muscles in patients with temporomandibular disorder. The patients were diagnosed with muscular TMD by clinical examination, and occlusal splints were inserted in all patients with a weekly follow-up. The superficial thermography on both sides of the muscles was performed using a digital thermometer in a controlled temperature room. This procedure was performed before occlusal splint insertion and after the completion of the treatment. After occlusal splint treatment, a significant increase in temperature was observed in each muscle, both on the right and left sides. When the muscles were compared in the same period of treatment, there was no significant difference among them which shows that the use of occlusal splint promoted a significant increase in the muscles temperature and that there was symmetry in the temperature of muscles on the right and left sides both before and after the treatment. The authors concluded that the results of the research are useful from the clinical point of view, and they show that thermographic visualization is beneficial not only in determining both activity and progress of the disease, which is a very similar conclusion as in previously mentioned articles [53, 54], but also in monitoring the progress of the treatment [31]. Barão VA et al. tested patients being treated for TMD by occlusal splints. Each patient was examined before treatment, and each temperature analysis was performed twice in each measurement time, two times before occlusal splint treatment and two times after treatment. A mean value of the two measurements was calculated. In all temperature measurements, the thermometer was positioned at 10 mm from each muscle surface. Thermography was performed before and after occlusal splint treatment. The follow-up of patients was 3.2 ± 1.01 months. Thermography was a sufficient tool to state a conclusion that occlusal splint therapy statistically increased the temperature of three parts of the masseter muscle (inferior, intermediate, and superior), anterior temporal, digastric, and trapezius muscles in patients with muscular temporomandibular disorder and also that there was a symmetry in the temperature of muscles on the right and left sides both before and after the treatment. The possibility of monitoring the progress of the treatment by using thermovision confirmed the research of de Melo et al. after years of research. Importantly, with the help of thermography, it can be visualized which muscles have an elevated temperature in the course of TMD, which can be used, among others, for treatment planning, including orthodontic treatment. The specific jaw setting through orthodontic treatment will affect selected muscles and muscle groups, and thus, the therapy method can be optimized based on imaging data so as to most effectively affect those muscles or their fragments whose temperature is the highest [55].

The study by Dibai-Filho et al. [56] aimed at assessing the correlation between TMD severity and the skin temperature above the temporomandibular joint (TMJ), masseter muscles, and anterior temporal muscle fibers. Cross-sectional studies were performed on a large group of patients, 60 women aged 18–40 years. The patients were assigned to groups based on the Fonseca Anamnestic Index (FAI): no TMD, mild TMD, moderate TMD, and severe TMD (*n* = 15 each). For each patient, the skin temperatures in the joint area, the masseter muscle, and the anterior temporal muscle were identified. It was found that the temperatures within the joint were statistically significantly higher within the group of patients with severe TMD symptoms. A similar group of patients consisting also only of women was examined by Haddad et al. [57]. The study conducted measurement of the cutaneous temperature of selected masticatory muscle regions of volunteers with and without myogenous temporomandibular disorder (TMD), using infrared thermography. The temperature levels measured at the masseter and anterior temporalis muscle regions in myogenous TMD volunteers were surprisingly significantly lower than those measured in controls, which is quite the opposite of all other presented research. The sensitivity and specificity of the thermographic assessment for the masseter region were 70% and 73%, respectively, and for the anterior temporalis region were 80% and 62%, respectively, but the study group was very small, the research was treated as a small-scale preliminary study, and therefore, to confirm these puzzling conclusions, it would be necessary to carry out the same research methodology on a much larger group of respondents, thus enabling the drawing of reliable, statistically significant conclusions.

A study of Magalhães et al. [58] noticed that joints and muscle disorders assessment and diagnosis methods require palpation or the application of certain forces on the skin, which affects the structures beneath which can be a possible influence on skin temperature. The aim of the experiment was to determine the ideal time for performing thermographic examination after palpation based on the assessment of skin temperature evolution. They concluded that infrared thermography can be used after assessment or diagnosis methods focused on the application of forces on tendons and muscles, provided the procedure is performed 15 minutes after contact with the skin. Regarding the myofascial trigger point, the thermographic examination can be performed within 60 minutes after the contact with the skin.

## 3. Conclusions

In the last 5 years, only a few studies using a thermal imaging camera in the diagnosis of temporomandibular joint dysfunction have been published. This is probably due to the difficulties in the interpretation of thermograms, which require specialized, dedicated analysis and processing of the obtained images. There is no standardized protocol for measuring masticatory muscle temperature using infrared imaging. This causes difficulties in implementing thermovision analysis as a standard diagnostic clinical procedure. To date, there are no objectified computer analytical tools, which would enable doctors to draw conclusions from the obtained images. Among the presented papers, only a few included sufficiently large study groups [34, 56], which made it possible to obtain statistically significant results. In the next two papers [41, 42], the authors studied individual cases, so they can be treated only as an indication of the problem for other researchers.

Importance should be accorded also regarding the choice of the selected software, in order to delimit an accurate, representative area of the region in question. Rodrigues-Bigaton et al. [30] used linear tools and a square area positioned along the masseter and anterior temporal muscles in order to check the mean temperature and correlation with regard to the diagnosis of myogenic TMD; however, none of the analytical methods was consistent and satisfactory [56].

Regardless of the analytical difficulties encountered, the described diagnostic method is worth further development. As a noninvasive technique, it does not pose any danger during in vivo tests and requires mainly patience and time from the person performing the test and the examined subject. It may result in minimizing the costs of measuring equipment, introducing it to dental offices and orienting it towards a standard screening tool for the occurrence of temporomandibular joint dysfunction [30, 57–65].

Further interest in thermography should be expected, primarily due to its zero invasiveness, speed of testing, and continuous development of thermal imaging cameras. Not only a sharp increase in the spatial resolution of cameras is observed which enables the possibility of imaging even with a resolution of 1080p but also a thermal resolution of even 0.001 K. In addition, more and more cameras allow for hybridization of the image in visible and infrared light, which allows precise identification of anatomical structures affected by inflammation. Hyperspectral thermal imaging cameras can be mentioned as a very interesting direction of thermal imaging cameras development. Hyperspectral cameras recording the spectrum in a wide wave range can be used to identify very narrow spectral ranges for individuals, perhaps not only to assess the skin temperature on its surface but also by using appropriate algorithms for analysis and processing of temperature assessment images in 3D of inflamed tissues [21, 66, 67].

However, attention should also be paid to the limitations of the thermovision method. These are primarily the need to control the measurement conditions, difficulties with complete objectification of the results—the impact of factors such as the patient's emotional state on the results obtained, and the lack of analytical tools that would allow for repetitive, fast, and objective analysis of data by a doctor in a dental office environment.

Nevertheless, despite these limitations, thermographic imaging can be a very interesting and useful tool in the diagnosis and assessment of the progress of TMD therapy.
